# Capsanthin supplementation modulates the immune response in broiler chickens under *Escherichia coli* lipopolysaccharide challenge

**DOI:** 10.5194/aab-66-103-2023

**Published:** 2023-03-02

**Authors:** Brigitta Csernus, Csaba Szabó, Renáta Knop, Reda Gebrehaweria Kidane, Sawadi Fransisco Ndunguru, Gabriella Gulyás, Xénia Erika Ozsváth, Levente Czeglédi

**Affiliations:** 1 Department of Evolutionary Zoology and Human Biology, University of Debrecen, Debrecen, 4032, Hungary; 2 Department of Animal Nutrition and Physiology, Institute of Animal Science, Biotechnology and Nature Conservation, Faculty of Agricultural and Food Sciences and Environmental Management, University of Debrecen, Debrecen, 4032, Hungary; 3 Department of Animal Science, Institute of Animal Science, Biotechnology and Nature Conservation, Faculty of Agricultural and Food Sciences and Environmental Management, University of Debrecen, Debrecen, 4032, Hungary; 4 Doctoral School of Animal Science, University of Debrecen, Debrecen, 4032, Hungary

## Abstract

Due to the legislation of antibiotic usage, natural substances are required for application in the poultry industry. Because of their potential anti-inflammatory and immunomodulatory effects, carotenoids are great sources. Capsanthin, a major carotenoid giving the red color of pepper, is a promising feed additive, as it can reduce chronic inflammation. This study was conducted to determine the effects of capsanthin supplementation at 80 mg kg ${}^{-1}$ in feed on the immune response of broiler chickens under *Escherichia coli* O55:B5 lipopolysaccharide (LPS) challenge. Ross 308 male broilers were divided into treatments: control (basal diet) and feed-supplemented groups. At 42 d of age, chickens were weighed and then challenged with 1 mg LPS per kilogram of body weight intraperitoneally. Four hours after injection, birds were euthanized, and then spleen and blood samples were collected. Capsanthin supplement at 80 mg kg ${}^{-1}$ did not change the growth parameters and the relative spleen weight. LPS immunization resulted in higher splenic interleukin-1 β (IL-1 β ), interleukin-6 (IL-6), and interferon- γ (IFN- γ ) mRNA expressions. Capsanthin addition reached lower gene expression levels of IL-6 and IFN- γ compared to the LPS-injected birds. At plasma level, dietary capsanthin resulted in lower IL-1 β and IL-6 levels. These results may indicate the potential anti-inflammatory effect of capsanthin supplementation in broiler chickens.

## 1 Introduction

Inadequate usage of antibiotics in poultry is receiving great attention due to the potential residues in chicken meat and antimicrobial resistance among bacterial populations (Abd El-Hack et al., 2020). Accordingly, the application of antibiotics is being regulated, and natural substances such as plant extracts have appeared in recent years (Alagawany et al., 2018). Among the plant substances, carotenoids are often used in poultry feed as a means of pigmentation of animal products, last but not least for their anti-inflammatory, antioxidant, and immunomodulatory effects (Marounek and Pebriansyah, 2018; Nabi et al., 2020). The anti-inflammatory effects of some carotenoid compounds, such as astaxanthin or other xanthophylls (lutein, zeaxanthin), have been proven (Lee et al., 2003; Gao et al., 2012). However, other potential carotenoid agents can be applied in feed to improve immune responses during inflammatory reactions. Capsanthin is one of the major carotenoids of red pepper. It comprises up to 60 % of the total carotenoids in pepper, but the proportion can vary among cultivars in the genus *Capsicum* (Perez-Galvez et al., 2003; Suzuki-Mori, 2003). Capsanthin is fat-soluble and has a molecular structure with a long chain of conjugated double bonds ending in one or two polar ketones. It absorbs green light to give a red–orange shade (Shah et al., 2014). The mentioned compound has an important role in animal nutrition, health, and reproduction, and it can ameliorate chronic inflammation and has a higher antioxidant activity than other xanthophylls (Shah et al., 2014; Perez-Galvez and Minguez-Mosquera, 2002). It could reduce the oxidative stress and the inflammation and inhibit the expressions of inflammatory cytokines, tumor necrosis factor- α (TNF- α ), pro-inflammatory mediators, and interleukins (IL-2, IL-4, IL-6) in rats (Shanmugham and Subban, 2022). Due to the very limited studies with capsanthin in birds, we aimed to investigate the effect of capsanthin on growth performance, relative spleen weight, and immune-related gene expression in broiler chickens under lipopolysaccharide (LPS) challenge. LPS is an integral component of the outer membrane of Gram-negative bacteria and causes acute or systemic inflammation (Akira et al., 2001; Guo et al., 2022). Therefore, it is a widely used model to study stress or inflammatory responses in broiler chickens (Lee et al., 2017; Chen et al., 2018). LPS can increase the degree of lipid peroxidation by elevating malondialdehyde (MDA) levels and decreasing the concentration of superoxide dismutase (SOD) antioxidant enzymes in male broilers at 42 d of age. In addition, LPS can enhance the gene expression levels of pro-inflammatory cytokines, such as interleukin-1 β and interferon- γ at the same age of male chickens (Zhang et al., 2021). Among the immune-related genes, interleukin-1 β (IL-1 β ), interleukin-6 (IL-6), interferon- γ (IFN- γ ), and toll-like receptor 4 (TLR-4) were examined in our study. As part of the innate and adaptive immunity, cytokines have signaling roles between cells and take part in cellular immune responses. Both interleukins (IL-1 β , IL-6) are pro-inflammatory ones (Dinarello, 2000; Kambayashi et al., 1995). IL-1 β stimulates macrophages and has a role in inflammatory reactions, and it activates T cells (Lotz et al., 1988; Klasing, 1988), while the higher level of IL-6 can be attributed to an acute-phase reaction (Hong et al., 2006). IFN- γ is a multifunctional pro-inflammatory cytokine originally known to interfere with viral replication; however, it has several roles during immune responses, such as stimulating the bactericidal activity of phagocytes (Oladele et al., 2018). TLR-4 is a transmembrane protein that recognizes the presence of LPS (Akira et al, 2001). In this study, IL-1 β and IL-6 cytokines were investigated at plasma protein level and, being part of the humoral immune response, immunoglobulin G (IgG) was also examined (Kaiser and Stäheli, 2008).

## 2 Materials and methods

### 2.1 Experimental design and growth parameters

Ross 308 male broilers were hatched at a local hatchery (Master Good Ltd., Petnehaza, Hungary), and a trial was conducted at the University of Debrecen, Institute for Agricultural Research and Educational Farm, Animal Husbandry Experimental Station (Kismacs, Debrecen, Hungary). All broilers were placed in the same room and kept in cages covered by card boxes individually. Temperature and light were provided according to the Aviagen Ross Management Handbook. Broiler chickens were randomly divided into treatments. The experiment started at 35 d of age and lasted until 42 d of age, since physiological, stress, or immunological parameters are often studied at this latter age (Liu et al., 2015; Zheng et al., 2020). The experimental diets consisted of the control group (basal diet) and capsanthin supplementation at 80 mg kg ${}^{-1}$ in feed. This is the maximum dosage at which capsanthin can be used (alone or in combination with other carotenoids or xanthophylls) as a colorant in poultry feed (except turkey) without any time limit established by the European Union Register of Feed Additives pursuant to Regulation (EC) No. 1831/2003 of Council Directive 70/524/EEC (European Food Safety Authority, 2020). Capsanthin was commercially available (ab142638, Abcam, Cambridge, UK) and dissolved in sunflower oil and then sprayed onto the basal diet during mixing. The basal diets (finisher) (Table 1) were corn–soybean meal diets and fed in mashed form. Broilers had free access to feed and water. On the last day of the trial, broiler chickens 42 d of age were weighed, and then daily gain and feed intake were determined. On the same day, six male broilers per treatment were injected with 1 mg kg ${}^{-1}$ body weight *Escherichia coli* O55:B5 LPS (L2880, Sigma, St. Louis, MO, USA) intraperitoneally (Takahashi et al., 2013). LPS was dissolved in sterile isotonic saline solution (B. Braun, Budapest, Hungary) to provide a concentration of 1 mg mL ${}^{-1}$ . Another six male chickens in the control group were also inoculated with 1 mL kg ${}^{-1}$ body weight saline solution (as the LPS vehicle in equivalent volume) in the same way.

**Table 1:** The composition and nutrient level of the basal (finisher) diet.

Basal ingredients	Value
Corn, %	32
Wheat, %	32
Soybean meal, solvent extracted (46.0 % CP), %	16
Soybean meal, extruded (46.0 % CP), %	4
Sunflower meal, extracted, %	4
Feed yeast, %	
DDGS, %	5
Plant fats, %	4
Premix, %	3
Total, %	100
Nutrient level	
Dry matter, %	89.15
AME ${}_{n}$ poultry, MJ kg ${}^{-1}$	13.01
Crude protein, %	18.28
Crude fat, %	6.83
Crude fiber, %	3.88
Lysine, %	1.09
Methionine, %	0.49
Methionine + cysteine, %	0.83
Calcium, %	0.67
Phosphorus, %	0.49

CP: crude protein. DDGS: distillers dried grains with solubles. AME ${}_{n}$ poultry: apparent metabolizable energy corrected for nitrogen.

### 2.2 Sample collection and lymphoid organ weight

Four hours later all of the injected birds were sacrificed by cervical dislocation. Blood was collected and separated into plasma by centrifugation at 3000 RCF (relative centrifugal force) and for 10 min. The whole spleen was aseptically excised and measured and then transferred to liquid nitrogen and stored at - 80 ${}^{\circ }$ C for RNA isolation. The relative spleen weight was calculated as follows: spleen weight divided by live weight and multiplied by 100 (Sławińska et al., 2014).

### 2.3 Gene expression measurement

Isolation of total RNA was carried out from spleen samples using DirectZol™ RNA MiniPrep (Zymo Research, Orange, CA, USA) according to the manufacturer's protocol. The yield of the obtained RNA was calculated by applying the Biotek Synergy HTX Multimode Reader (Agilent Technologies, Inc., Santa Clara, CA, USA). RNA integrity was checked by 1 % agarose gel electrophoresis. The cDNA synthesis was performed from 200 ng of total RNA using qScript^®^ cDNA supermix (Quantabio, Beverly, MA, USA) according to the manufacturer's protocol. cDNA samples were diluted 10-fold and stored at - 20 ${}^{\circ }$ C. Primer pairs for chicken target genes (IL-1 β , IL-6, IFN- γ , and TLR-4) were designed as reported in our previous study (Csernus et al., 2020). The primer details are listed in Table 2. Real-time PCR was carried out in an Agilent AriaMx real-time PCR (Agilent Technologies, Inc., Santa Clara, CA, USA). Reactions were run in duplicates using a 96-well plate (FrameStar, 4titude, Surrey, UK). Each reaction included 2 ng cDNA, 5 × HOT FIREPol^®^ EvaGreen^®^ qPCR Mix Plus (ROX) (Solis BioDyne, Tartu, Estonia), and 200 nM of each primer and distilled water in a 10 µ L final volume. No template controls were involved for each gene. RT(real-time)-PCR conditions were the following: initial denaturation at 90 ${}^{\circ }$ C for 12 min, 40 cycles of denaturation at 90 ${}^{\circ }$ C for 2 min, primer annealing at 60 ${}^{\circ }$ C for 20 s, and elongation at 72 ${}^{\circ }$ C for 20 s. Ct values and melting curves were collected by AriaMx 1.8 software. In the spleen, GAPDH was considered the most stable gene for normalization by the NormFinder, Best Keeper, and Δ Ct methods. Results were calculated using the 2 ${}^{\Delta \Delta }$ Ct relative quantitative method (Livak and Schmittgen, 2001). Relative expressions were determined as fold changes in the expression of the target gene in the treatment group compared with the LPS-injected control group.

**Table 2:** Primer sequences of immune-related genes.

Gene	Gene name	GenBank	Primer sequences	Amplicon	Tm
symbol		accession no.	( $5^{\prime }\to 3^{\prime }$ )	length	( ${}^{\circ }$ C)
IL-1 β	Interleukin-1 β	XM_015297469.1	F: TGCTTCGTGCTGGAGTCACCC	98	59.93
			R: GGCCGGTACAGCGCAATGTT		59.02
IL-6	Interleukin-6	XM_015281283.2	F: AGCGAAAAGCAGAACGTCGAGTC	107	58.73
			R: GCCGAGTCTGGGATGACCACTTC		59.94
IFN- γ	Interferon- γ	NM_205149.1	F: AACAACCTTCCTGATGGCGTGA	89	57.46
			R: GCTTTGCGCTGGATTCTCAAGT		57.02
TLR-4	Toll-like receptor 4	NM_001030693.1	F: ACCCGAACTGCAGTTTCTGGAT	120	57.2
			R: AGGTGCTGGAGTGAATTGGC		57.61

### 2.4 Enzyme-linked immunosorbent assay (ELISA)

Chicken plasma samples were used to evaluate IL-1 β , IL-6, and IgG levels with an ELISA kit (Xinqidi, Wuhan, China). ELISA kits followed the sandwich ELISA technique. Blanks, standards, and samples were involved in duplicates. Based on the kit specifications, the absorbance was determined at 450 nm using the Biotek Synergy HTX Multimode Reader (Agilent Technologies, Inc., Santa Clara, CA, USA). Protein concentrations were calculated using the equation from the linear regression of the obtained standard curve.

### 2.5 Statistical analysis

The main effect of capsanthin treatment under LPS challenge was analyzed using a one-way analysis of variance (one-way ANOVA) Tukey test by GraphPad Prism 8.0.1 software. Differences among treatments were considered significant at P<0.05 . Results were presented as the mean ± standard error of the mean (SEM).

## 3 Results

### 3.1 Growth parameters

Our results showed that capsanthin supplementation at a concentration of 80 mg kg ${}^{-1}$ in feed did not affect the growth performance (Table 3) of broiler chickens ( P>0.05 ).

**Table 3:** Effect of dietary capsanthin supplementation on growth parameters of broiler chickens at 42 d of age.

Growth performance	Control	Capsanthin	P value
Body weight, g	2742 ± 60.18	2734 ± 103.2	0.9454
Body weight gain, g d ${}^{-1}$	86 ± 4.504	73 ± 9.293	0.1709
Feed intake, g d ${}^{-1}$	184 ± 2.638	183 ± 3.052	0.8576

Control: control group ( n=12 ). Capsanthin: dietary capsanthin supplement at 80 mg kg ${}^{-1}$ in feed ( n=6 ). P<0.05 . A significant difference was not observed between the treatment groups.

### 3.2 Relative spleen weight and immune-related gene expression

**Figure 1:**
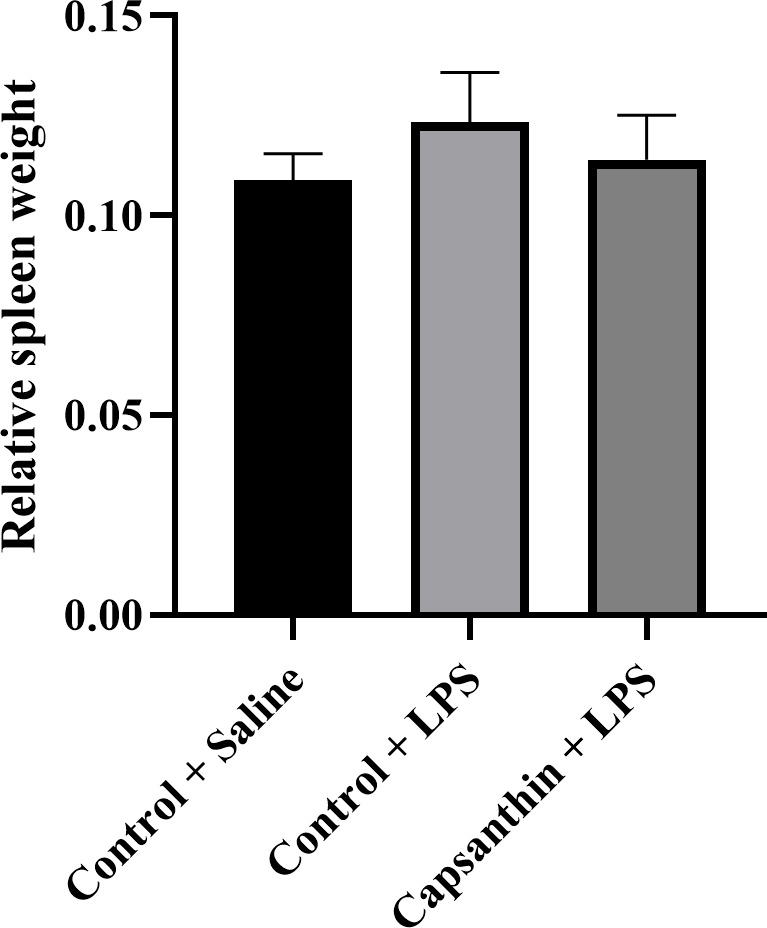
Relative spleen weight of broiler chickens. Control + saline: chickens fed a basal diet with isotonic saline injection. Control + LPS: chickens fed a basal diet under *E. coli* O55:B5 LPS challenge. Capsanthin + LPS: chickens fed a diet supplemented with capsanthin at 80 mg kg ${}^{-1}$ under *E. coli* O55:B5 LPS challenge ( n=6/ treatment). Data are presented as means ± standard errors of the mean. The impact was analyzed by one-way ANOVA, and differences among the groups were considered significant at P<0.05 . The effect of dietary supplementation was not significant.

Relative spleen weight did not show significant differences ( P>0.05 ) among the groups (Fig. 1). Results were 0.108 % in control (saline) birds, 0.123 % in the control (LPS) group, and 0.114 % in the capsanthin (LPS) treatment.

**Figure 2:**
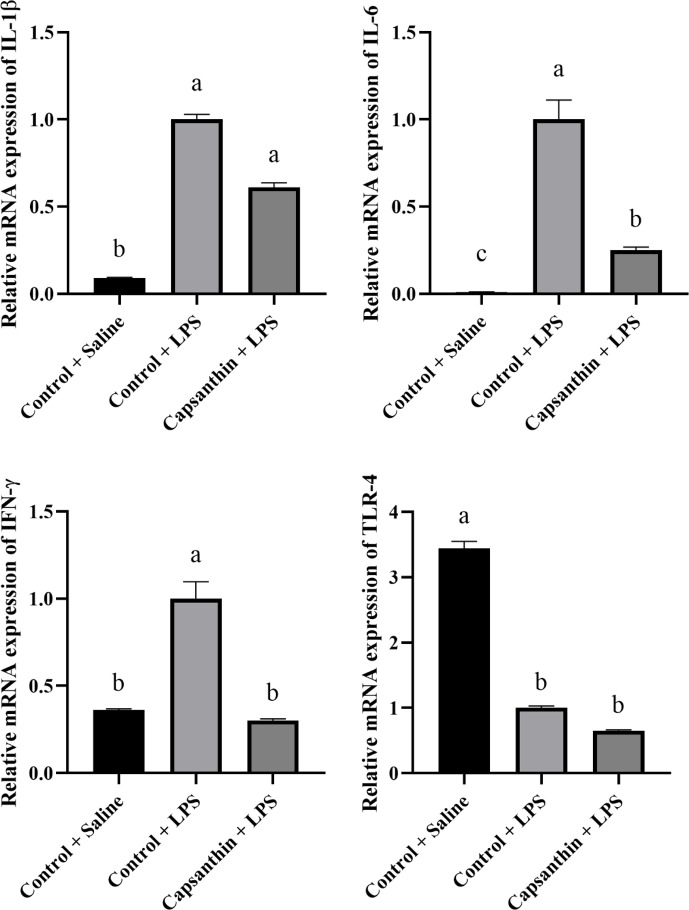
Relative mRNA levels of splenic interleukin-1 β (IL-1 β ), interleukin-6 (IL-6), interferon- γ (IFN- γ ), and toll-like receptor 4 (TLR-4) in broiler chickens. Control + saline: chickens fed a basal diet with isotonic saline injection. Control + LPS: chickens fed a basal diet under *E. coli* O55:B5 LPS challenge. Capsanthin + LPS: chickens fed a diet supplemented with capsanthin at 80 mg kg ${}^{-1}$ under *E. coli* O55:B5 LPS challenge ( n=6/ treatment). Data are presented as means ± standard errors of the mean. The impact was analyzed by one-way ANOVA, and differences among the groups were considered significant at P<0.05 . Means with a, b, and c differ significantly at P<0.05 .

Relative mRNA expressions of IL-1 β , IL-6, IFN- γ , and TLR-4 are shown in Fig. 2. The gene expression level of the pro-inflammatory IL-1 β was higher ( P=0.0002 ) in the control (LPS) group compared to the saline-injected control group. Capsanthin supplementation at 80 mg kg ${}^{-1}$ in feed did not result in a lower gene expression level of IL-1 β compared to the LPS-injected birds. The gene expression level of the pro-inflammatory IL-6 was also higher ( P<0.0001 ) in the LPS-injected control birds compared to the control (saline) ones. Capsanthin addition at 80 mg kg ${}^{-1}$ decreased the level of IL-6 compared to the LPS group. The relative mRNA expression of IFN- γ was higher in the LPS-treated birds, and capsanthin supplementation eventuated in a lower ( P=0.0382 ) mRNA level of the pro-inflammatory IFN- γ . Relative mRNA levels of TLR-4 were lower in the control (LPS) ( P=0.0351 ) and capsanthin (LPS) ( P=0.0023 ) groups compared to control (saline) birds.

### 3.3 Plasma IL-1 β , IL-6, and IgG concentrations 

**Figure 3:**
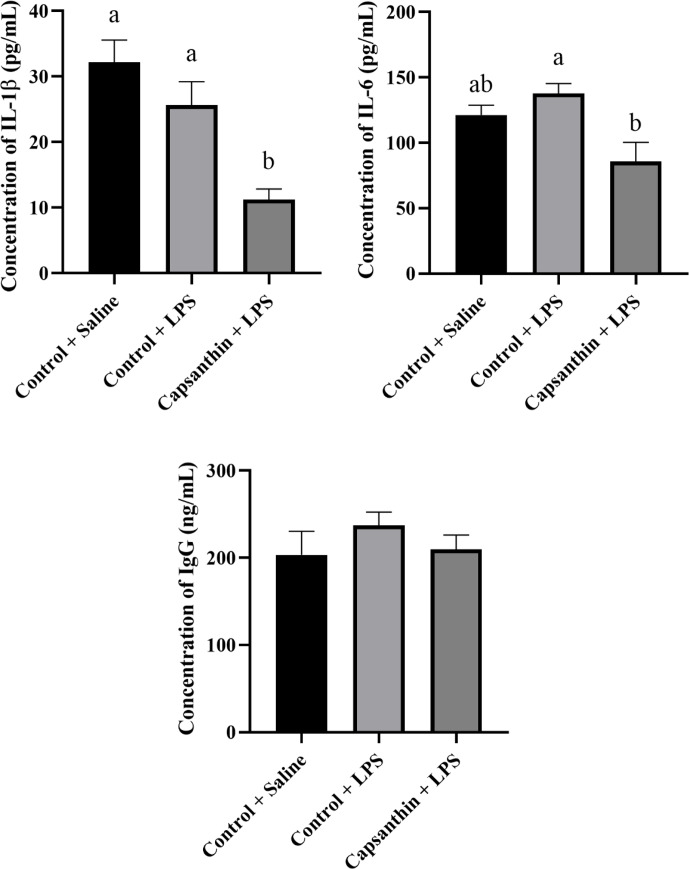
Concentrations of plasma interleukin-1 β (IL-1 β ), interleukin-6 (IL-6), and immunoglobulin G (IgG) of broiler chickens. Control + saline: chickens fed a basal diet with isotonic saline injection. Control + LPS: chickens fed a basal diet under *E. coli* O55:B5 LPS challenge. Capsanthin + LPS: chickens fed a diet supplemented with capsanthin at 80 mg kg ${}^{-1}$ under *E. coli* O55:B5 LPS challenge ( n=6/ treatment). Data are presented as means ± standard errors of the mean. The impact was analyzed by one-way ANOVA, and differences among the groups were considered significant at P<0.05 . Means with a and b differ significantly at P<0.05 .

Plasma concentrations of IL-1 β , IL-6, and IgG are shown in Fig. 3. IL-1 β levels were 32.19, 25.62, and 11.24 pg mL ${}^{-1}$ in the control (saline), control (LPS), and capsanthin (LPS) groups, respectively. Concentrations of IL-1 β did not differ among the control (LPS) and control (saline) groups. Capsanthin supplementation decreased ( P=0.0496 ) the level of the mentioned pro-inflammatory cytokine compared to the LPS-injected birds. The concentration of pro-inflammatory IL-6 was 121.2, 137.8, and 85.79 pg mL ${}^{-1}$ in the control (saline), control (LPS), and capsanthin (LPS) groups, respectively. IL-6 concentrations did not differ significantly between the control groups (LPS and saline). However, capsanthin supplementation could reach a lower level ( P=0.0263 ) of IL-6 compared to LPS-injected chickens. Plasma IgG concentrations were 203.3, 237.2, and 209.7 ng mL ${}^{-1}$ in the control (saline), control (LPS), and capsanthin (LPS) treatments, respectively. IgG levels did not differ significantly among the groups.

## 4 Discussion

A short experiment was conducted to investigate the effect of capsanthin supplementation on growth performance and immune response under *Escherichia coli* O55:B5 LPS challenge. The effect of feed supplemented with capsanthin at 80 mg kg ${}^{-1}$ was examined on growth parameters, such as body weight, body weight gain, and feed intake. Due to the limited number of chickens ( n=12/ control and n=6/ treatment), performance parameters were calculated as background information to support the immunological study as the core part of the trial. None of the mentioned traits was affected by the supplementation. In contrast, chili pepper powder addition at 0.5 % and 1 % in the feed had positive effects on the body weight of chickens at 35 and 42 d of age (Puvača et al., 2019). Al-Kassie et al. (2011) reported the same, and body weight and feed conversion ratio were improved by chili pepper treatments at levels of 0.5 %, 0.75 %, and 1 % in the feed of broiler chickens. Thiamhirunsopit et al. (2014) also noted improved growth performance results after different chili pepper treatments compared to control birds.

The effect of capsanthin supplementation on immune response in broiler chickens under *E. coli* LPS challenge was also examined. LPS immunization induces an acute inflammatory response and stimulates the synthesis of pro-inflammatory cytokines in broilers. In this research, LPS inoculation was applied in both the capsanthin-treated and control groups to evoke immune response and to evaluate the impact of the mentioned carotenoid on immunological parameters during inflammatory responses. Saline inoculation was used as a vehicle in equivalent volume in the control group, and the impact of capsanthin was not examined without causing inflammation.

In our study, the relative spleen weights were not changed among the treatments. In contrast, the relative weight of this lymphoid organ was higher in the LPS-injected broilers compared to the lutein-supplemented, saline-injected birds (Rajput et al., 2013). Koutsos et al. (2006) reported higher spleen weights for the non-supplemented birds under LPS challenge, in contrast to the lutein-treated birds, and the authors discussed severe systemic inflammatory response after LPS injection for the non-treated birds. In this study, immune-related gene expression analysis was carried out to investigate the effect of capsanthin in broiler chickens under LPS immunization. The relative mRNA level of IL-1 β was higher in the LPS-treated control group compared to the saline-injected control birds. Similarly, gene expression levels of splenic and ileal IL-1 β were also increased in the LPS-injected control birds (Wu et al., 2017). Therefore, *E. coli* lipopolysaccharides induced an acute-phase response and a bacterial illness, which was also confirmed in our previous study (Csernus et al., 2020). Capsanthin supplementation decreased the mRNA expression of the mentioned interleukin. Similarly, splenic IL-1 β was decreased in chickens by dried-scent leaf meal (rich in carotenoids), and the authors discussed that the dried-scent leaf meal may regulate the inflammation in chickens and may be used as a replacement for in-feed antibiotics in chicken production (Sorhue et al., 2021).

In our study, the gene expression level of pro-inflammatory IL-6 was elevated in the LPS-injected birds in contrast to the saline-inoculated ones, which can be discussed as an acute-phase reaction (Hong et al., 2006). Capsanthin supplementation reduced the mRNA level of the cytokine. Meriwether et al. (2010) reported the same, and splenic IL-6 mRNA abundance was higher in the LPS-injected laying chickens, in contrast to the control (non-vaccinated) ones. The authors also applied lutein (a carotenoid source) at a concentration of 40 mg kg ${}^{-1}$ in the diet of laying chicks through 30 d, resulting in carotenoid-replete eggs. Twelve days after hatching, an inflammatory challenge (LPS) was used. As a result of in ovo carotenoid exposure, the mRNA level of IL-6 could decrease compared to those chickens hatched from carotenoid-deplete eggs, and as discussed, lutein can decrease the inflammatory parameters in the spleen (Meriwether et al., 2010).

In this study, capsanthin influenced the level of pro-inflammatory IFN- γ as well, and the supplement at 80 mg kg ${}^{-1}$ reached a lower gene expression level of the mentioned interferon. Similarly, the relative mRNA level of IFN- γ was inhibited in the liver and jejunum of chickens without any challenge on experimental day 35, when dietary xanthophylls were fed at 40 mg kg ${}^{-1}$ (Gao et al., 2012). Pourabedin et al. (2017) noted the same, and feed supplementation reduced the level of IFN- γ cecal tonsils of chickens under *Salmonella enteritidis* challenge. In the same study, the authors concluded that IFN- γ takes part in macrophage activation and nitric oxide production.

TLR-4 is an important receptor of LPS and stimulates the secretion of pro-inflammatory cytokines, which are crucial for enhancing potent immune responses (Gorina et al., 2011; Mateu et al., 2015). In our study, the relative mRNA expression level of TLR-4 decreased in the LPS-challenged control group. Since TLR-4 recognizes LPS, the opposite was expected. Similarly to our results, Guo et al. (2022) noted the same, and the gene expression level of TLR-4 was lower in the LPS-injected broiler chickens when LPS was applied at the same concentration (1 mg kg ${}^{-1}$ BW). The authors discussed that chickens may respond differently to LPS compared to mammals, and the responding patterns may be attributed to the diverse species and LPS dosages. The gene expression level of TLR-4 was inhibited in capsanthin-fed birds as well. Cheng et al. (2017) reported the same when baicalin (a flavonoid compound) at concentrations of 50, 100, and 200 mg kg ${}^{-1}$ in feed decreased the gene expression levels of TLR-4 and concluded that the flavonoid could alleviate LPS-induced inflammatory responses in chickens and have an anti-inflammatory effect.

The impact of capsanthin supplementation on plasma concentrations of IL-1 β , IL-6, and IgG was also determined in this study. In contrast to the relative mRNA expressions in spleen, plasma protein levels of IL-1 β and IL-6 did not change in the LPS-injected control group compared to the saline-inoculated control birds, which could be due to the post-transcriptional phenomena, such as the mRNA stability or the elevated half life of protein resulting from the post-translational modifications often altering the protein levels (Ideker et al., 2001). However, capsanthin supplementation decreased plasma IL-1 β concentration compared to both control groups, which may indicate the potential anti-inflammatory property of the mentioned carotenoid. The plasma concentration of IL-6 was lower in the feed-supplemented group compared to the LPS-injected control birds, so capsanthin may decrease the level of inflammation at plasma level as well. The effect of capsanthin on plasma IgG concentration was also examined in this study; however, it did not change in the treated group. Zhou et al (2019) reported that feed supplementation resulted in elevated levels of jejunal and ileal IgG contents, and the authors discussed improved immune function. Cai et al. (2012) defined increased serum IgG levels in broiler chickens when feed supplementation was applied, which was explained with improved humoral immunity by the authors.

## 5 Conclusions

In conclusion, dietary capsanthin at the applied concentration could mostly affect the immune parameters of broiler chickens under *Escherichia coli* LPS challenge. Capsanthin supplementation at 80 mg kg ${}^{-1}$ in feed decreased the relative gene expression levels of IL-6 and IFN- γ and further reduced plasma IL-1 β and IL-6 concentrations compared to the LPS-injected birds. Therefore, the usage of capsanthin is being suggested to mitigate the inflammation during cellular immune response in broiler chickens.

## Data Availability

The original data of the paper are available upon request from the corresponding author.
